# Plasma *Plasmodium falciparum* Histidine-rich Protein 2 Concentrations in Children With Malaria Infections of Differing Severity in Kilifi, Kenya

**DOI:** 10.1093/cid/ciaa1141

**Published:** 2021-10-05

**Authors:** Sophie Uyoga, Perpetual Wanjiku, Jesse C. Rop, Johnstone Makale, Alexander W. Macharia, Gideon M. Nyutu, Mohammed Shebe, Kennedy A. Awuondo, Neema Mturi, Charles J. Woodrow, Arjen M. Dondorp, Kathryn Maitland, Thomas N. Williams

**Affiliations:** 1 KEMRI-Wellcome Trust Research Programme, Kilifi, Kenya; 2 Mahidol-Oxford Research Unit, Faculty of Tropical Medicine, Mahidol University, Bangkok, Thailand; 3 Centre for Tropical Medicine and Global Health, Nuffield Department of Medicine, University of Oxford, United Kingdom; 4 Department of Infectious Diseases, Imperial College, London, United Kingdom

**Keywords:** malaria, *Plasmodium falciparum* histidine-rich protein-2, *Pf*HRP2, parasite biomass, sequestration

## Abstract

**Background:**

Most previous studies support a direct link between total parasite load and the clinical severity of *Plasmodium falciparum* malaria infections.

**Methods:**

We estimated *P falciparum* parasite loads in 3 groups of children with malaria infections of differing severity: (1) children with World Health Organization–defined severe malaria (n = 1544), (2) children admitted with malaria but without features of severity (n = 200), and (3) children in the community with asymptomatic parasitemia (n = 33).

**Results:**

Peripheral parasitemias were highest in those with uncomplicated malaria (geometric mean [GM] parasite count, 111 064/μL; 95% confidence interval, CI, 86 798–141 819/μL), almost 3 times higher than in those with severe malaria (39 588/μL; 34 990–44 791/μL) and >100 times higher than in those with asymptomatic malaria (1092/μL; 523–2280/μL). However, the GM *P. falciparum* histidine-rich protein 2 (*Pf*HRP2) values (95% CI) increased with severity, being 7 (4–12) ng/mL in asymptomatic malaria, 843 (655–1084) ng/mL in uncomplicated malaria, and 1369 (1244–1506) ng/mL in severe malaria. *Pf*HRP2 concentrations were markedly lower in the subgroup of patients with severe malaria and concomitant invasive bacterial infections of blood or cerebrospinal fluid (GM concentration, 312 ng/mL; 95% CI, 175–557 ng/mL; *P* < .001) than in those without such infections (1439 ng/mL; 1307–1584; *P* < .001).

**Conclusions:**

The clinical severity of malaria infections related strongly to the total burden of *P. falciparum* parasites. A quantitative test for plasma concentrations of *Pf*HRP2 could be useful in identifying children at the greatest clinical risk and identifying critically ill children in whom malaria is not the primary cause.

*Plasmodium falciparum* histidine-rich protein 2 (*Pf*HRP2) is a 30-kDa molecule that is produced by most strains of *P. falciparum* malaria parasites [1] and is involved in the conversion of the toxic molecule heme to the more neutral malaria pigment hemozoin [2]. About 5 fg of *Pf*HRP2 is released from the cytoplasm of *P falciparum–infected* red blood cells during schizont rupture [3, 4], and *Pf*HRP2 can therefore be detected in the plasma of individuals with *P. falciparum* infection by means of enzyme-linked immunosorbent assay or rapid diagnostic tests [5, 6]. Previous studies have shown that plasma *Pf*HRP2 provides a more accurate reflection of the total *P falciparum* load in individuals than parasite counts in peripheral blood because, unlike the latter, *Pf*HRP2 levels provide information about the burden of mature-phase parasites that are sequestered in the deep vasculature [7].

Positive associations have been reported between both plasma *Pf*HRP2 and disease severity in a number of previous studies [7–12]. However, this has not been seen universally [13, 14], prompting some to question the causal link between sequestration and severe malaria [15]. We have estimated the *P. falciparum* parasite loads in children with malaria infections of differing severity in Kilifi, Kenya, with the aim of contributing to this debate.

## Methods

### Study Design and Participants

Our study involved 3 groups of children <14 years of age who were residents of Kilifi County, Kenya: (1) children admitted to the High Dependency Unit at Kilifi County Hospital with ≥1 feature of severe falciparum malaria; (2) children admitted to the general pediatric ward with uncomplicated malaria; and (3) children from the surrounding area with asymptomatic malaria infections.

### Clinical and Laboratory Data

Data and samples from children in groups 1 and 2 were captured through a routine surveillance system that has operated at Kilifi County Hospital since 1989, as described in detail elsewhere [16]. Severe malaria was defined as *P. falciparum* blood-film positivity with ≥1 of the following clinical or laboratory features: cerebral malaria (Blantyre coma scale score <3), respiratory distress (abnormal deep breathing), severe malarial anemia (hemogloblin level <5 g/dL), or other features of severity, as described elsewhere [17, 18]. Children in group 1 were admitted between 1998 and 2010 and those in group 2 between 2004 and 2005; children in group 3 were recruited during cross-sectional community surveys conducted in 2010-2011 [19], based on the availability of archived samples.

All laboratory tests were conducted at the KEMRI-Wellcome Trust Research laboratories, which are accredited according to Good Clinical Laboratory Practice standards (assessment by Qualogy). Parasitemia was determined in real time from blood films using standard methods [20], and *Pf*HRP2 was batch-analyzed by enzyme-linked immunosorbent assay in plasma samples archived at −80^°^C since the time of collection [10]. Hemoglobin S (HbS) and α-thalassemia were genotyped by polymerase chain reaction as described elsewhere [21, 22].

### Parasite Burden

The total number of circulating parasites (*P*_circ_), the whole-body total parasite load (*P*_tot_), and the sequestration index (SI), an indication of the proportion of parasites that are hidden from detection through sequestration in deep vascular beds, were estimated for each participant individually using published formulas [7, 10], as follows:

(1) *P*_circ_ = Parasites/μL × 10^6^ × circulating blood volume [0.08 L/kg] × body weight [kg],
(2) *P*_tot_ = 7.3 × *Pf*HRP2 [g/L] × (1 – hematocrit) × body weight [kg] × 10^13^,

and

(3) SI = *P*_tot_/P_circ_.

### Statistical Analysis

Continuous data were compared using Student’s *t* or Mann-Whitney tests as appropriate, and proportions were compared using the χ^2^ test. Nonnormal data were log-transformed before analysis. Linear regression was performed both with and without adjustment for ethnicity, HbS phenotype and α-thalassemia genotype. Children displaying multiple features of severe malaria were included in multiple categories. All data were analyzed using Stata software, version 15.1 (StataCorp).

### Ethics

Written informed consent was provided by the parents of all participants. Ethical approval was granted by the Kenya Medical Research Institute/National Ethical Review Committee in Nairobi, Kenya, and the Oxford Tropical Research Ethics Committee in Oxford, United Kingdom.

## Results

The study flow is summarized in Supplementary Figure 1. Clinical and laboratory data and plasma samples from a total of 1544 children with severe malaria, 200 with uncomplicated malaria, and 33 with asymptomatic malaria were retrieved for this study. *Pf*HRP2 was undetectable in 30 (1.7%) of these samples (23 from patients with severe, 2 from patients with uncomplicated, and 5 from patients with asymptomatic malaria), leaving 1747 contributing data to the final analysis.

### Demographic and Clinical Features

**Figure 1:**
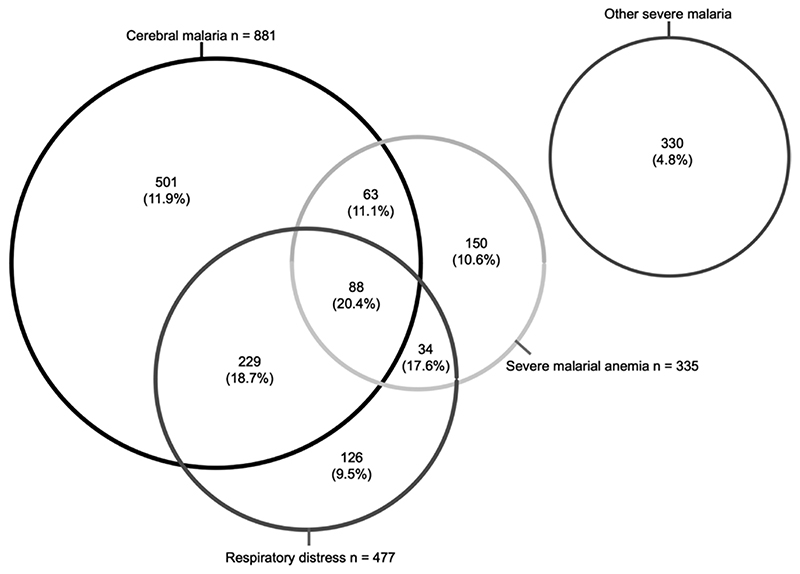
Distribution of clinical syndromes among patients with severe malaria. Numbers represent the number of children in each group, with in-hospital mortality rates in parentheses.

The demographic and clinical characteristics of participants are summarized in Table 1. Children with severe malaria were significantly younger than those with uncomplicated malaria, and those with asymptomatic malaria were significantly older. Overall, the in-hospital mortality rate was 11.7% in children admitted with severe malaria but 0% in those admitted with uncomplicated malaria. Many children with severe malaria presented with >1 severity feature (Figure 1). Mortality rates were similar (9.5%-11.1%) across the 3 main groups (cerebral malaria, respiratory distress, and severe malarial anemia) but were higher in children with 2 features and higher still (20.5%) in those with all 3 (*P* < .001). Conversely, the mortality rate was comparatively lower (4.8%) in those with none of the main features but who instead displayed other features such as prostration, hypoglycemia, and hyperparasitemia (Table 1).

### Markers of Parasite Biomass

Overall, parasite densities in peripheral blood were lowest in children with asymptomatic, highest in those with uncomplicated, and intermediate in those with severe malaria (Table 2). By contrast, a stepwise increase in plasma *Pf*HRP2 levels was seen between these 3 severity classes, with the highest (geometric mean [GM], 1369 ng/mL; 95% confidence interval [CI], 1244–1506 ng/mL) seen in those with severe malaria (Table 2).

**Figure 2:**
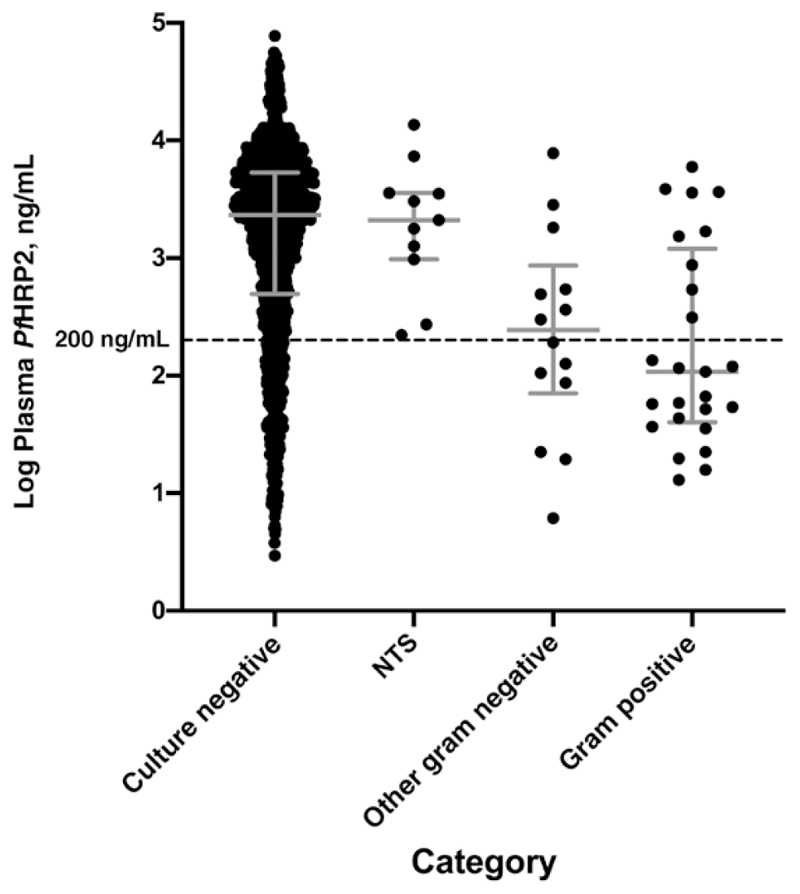
Plasma *Plasmodium falciparum* histidine-rich protein 2 (P/HRP2) levels in patients with severe malaria according to category of invasive bacterial infection. Error bars represents medians with interguartile range. Categories include nontyphoidal *Salmonella* species (NTS; n = 11); other gram-negative bacteria, including *Enterobactercloacae(n =* 1), *Escherichia coli(n =* 4), *Haemophilus influenzae* (n = 3), *Neisseria species* (n = 1), *Pseudomonas species* (n = 4) *Shigella sonnei* (n = 1); and gram-positive bacteria, including *Staphylococcus aureus* (n = 4), group A *Streptococcus* (n = 4), *Streptococcus pneumoniae* (n = 16), and *Streptococcus viridans* (n = 1).

One child with uncomplicated malaria and 50 with severe malaria had concomitant invasive bacterial infections (IBIs) diagnosed by culture of blood or cerebrospinal fluid (see Table 1 footnote for organisms and origins). Among patients with severe malaria, *Pf*HRP2 values were markedly lower in those with IBIs (GM, 312 ng/mL; 95% CI, 175–557 ng/mL) than in those without (1439 ng/mL; 1307–1584 ng/mL; *P* < .001). This difference was not seen in the subset of 11 infections with nontyphoidal *Salmonella* species (GM, 1883 ng/mL; 95% CI, 811–4371 ng/mL; *P* > .99), being confined to the 39 other gram-negative and gram-positive infections (188 ng/mL; 101-352 ng/mL; *P* < .001) (Figure 2 and Table 1). Of these 39 patients with *non-Salmonella* IBIs, 23 had a plasma *Pf*HRP2 value <200 ng/mL (a previously proposed cutoff for identifying patients at high risk of an alternative diagnosis), along with 240 patients without IBI. Hence 23 of 263 patients (8.7%) with a plasma *Pf*HRP2 level <200 ng/mL had an IBI, compared with only 16 of 1258 (1.3%) with levels above this threshold (an approximately 7-fold difference).

Plasma *Pf*HRP2 levels exceeded 1000 ng/mL in 1007 of 1521 children (66.2%) with severe malaria and 104 of 198 (52.5%) with uncomplicated malaria but was <1000 ng/mL (range, 0.9-91.3 ng/mL) in all children with asymptomatic malaria. The parameter that most markedly differed between groups was the SI. The GM SI (95% CI) was only 4.0 (1.8-8.8) and 4.9 (3.5-6.8) in children with asymptomatic or uncomplicated malaria, respectively, but was 40.6 (35.7-46.0) in those with severe malaria (*P* < .001). Overall, after adjustment for HbS, α-thalassemia, and ethnicity, the GM SI was 8.3 (95% CI, 5.6-12.3) times higher in children admitted with severe malaria than in those admitted with uncomplicated malaria. Within those with severe malaria only, parasite densities were significantly lower in the subgroup who died in the hospital, although a trend was seen toward an increased SI (Table 2).

### Markers of Parasite Biomass in Children With Different Phenotypes of Severe Malaria

Finally, we calculated the various measures of parasite biomass in children with specific severe malaria phenotypes and compared them with values from children in the uncomplicated group (Table 3). Among the children with severe malaria, peripheral parasite densities were lowest in those with none of the 3 major features and highest in those with respiratory distress. The most notable differences in *Pf*HRP2-based values related to children with severe malarial anemia, in whom GM plasma concentrations were 3.2 (95% CI, 2.3–4.5; *P* < .001) times higher and the SI 18.2 (11.8–28.2; *P* < .001) times higher than in children with uncomplicated malaria.

## Discussion

We have estimated both the peripheral and sequestered burdens of *P. falciparum* parasites in children with malaria of differing severity in Kilifi County, Kenya. We found no significant differences in the SI between those with asymptomatic and uncomplicated *P falciparum* malaria infections, but the SI was approximately 8 times higher in those with severe malaria, being particularly high in those with severe malarial anemia. Our study supports the hypothesis that malaria severity is proportionate to total parasite load, an observation that could be helpful in directing care to those at greatest need [5, 10].

A direct relationship between parasite load and malaria severity was first suggested in the late 19th century [23], and most studies that have investigated this question more recently through *Pf*HRP2-based methods have supported this general conclusion. In the first study of that kind, a strong relationship was seen with total parasite load in adults admitted to hospital on the Thai-Burmese border [7]. Loads were approximately 6 times higher in patients with severe than in those with nonsevere malaria and were especially high in those who later died. Similar results were found in a second study, in Indonesian adults [8].

Children presenting in coma with a positive malaria blood film result in Africa are generally assumed to have cerebral malaria. However, asymptomatic parasitemia is common during childhood, and one postmortem study showed that many such children have an alternative diagnosis [24]. Subsequently, Seydel and colleagues [25] demonstrated the potential utility of plasma *Pf*HRP2 concentration as a marker of intracerebral parasite sequestration, identifying children with cerebral malaria confirmed by either autopsy or malarial retinopathy. Later studies have confirmed the strong relationship between total parasite burden and malaria severity in African children [10, 26]. First, within the multinational AQUAMAT trial, high *Pf*HRP2 concentrations were found in the majority of children while levels were significantly higher in those who died than in those who survived. Of particular interest, a *U*-shaped association was seen between *Pf*HRP2 concentration and death, potentially explained by the misclassification to malaria of the subgroup with the lowest concentrations [10].

In a subsequent study conducted in Tanzanian children, the plasma *Pf*HRP2 level was 19 (15–23) ng/mL in asymptomatic carriers, 163 (137–194) ng/mL in children with uncomplicated malaria, detected in the community, and 1510 (1180–1933) and 1746 (1577–1934) ng/mL among children with severe malaria admitted during 2 different time periods [26]. A concentration of <200 ng/mL was found to indicate severe febrile illness caused by an alternative diagnosis in >10% of patients. Our data relating to IBIs extend these findings and exemplify the potential for plasma levels to contribute to clinical diagnosis. *Pf*HRP2 levels were substantially lower in the subgroup of children with severe malaria who also had a concomitant IBI.

As expected, this was not the case for the subset whose cultures were positive for nontyphoidal *Salmonella* species, because previous studies have shown that IBIs due to *Salmonella* can complicate malaria via a mechanism involving gut barrier dysfunction [27]. Compared with children without IBIs, plasma *Pf*HRP2 levels were approximately 7.5 times lower in children with other gram-negative as well as gram-positive bloodstream infections, indicating that these IBIs were likely to have been the main agents of severe illness in many of these children. In our study, plasma *Pf*HRP2 levels were <200 ng/mL in only one-sixth of severe cases but identified 23 of the 39 children with *non-Salmonella* IBIs: that is, approximately 9% of children with these low *Pf*HRP2 levels had an IBI. The 16 other children with such IBIs were among 1258 with a *Pf*HRP2 level above this cutoff (1.3%). Hence, the 200-ng/mL threshold enriched for children with non–malaria-associated IBIs by approximately 7-fold.

These findings are similar to those of Hendriksen and colleagues [26], although in their study the relationship between plasma *Pf*HRP2 concentration and distinct categories of IBI was less clear cut than in the current study. *Pf*HRP2 levels have also been correlated with childhood cerebral malaria in a number of smaller studies conducted in Tanzania [9], Malawi [11], and Uganda [12], and with various forms of adult malaria among imported cases in France [28]. At present, there are no point-of-care methods for quantifying *Pf*HRP2. The development of such tests in the future would be of major potential benefit.

The pathogenesis of severe falciparum malaria is complex, but a compromised microcirculation in vital organs caused by the sequestration of cytoadhered parasitized red blood cells to the vascular endothelium is central, compounded by endothelial dysfunction, reduced red blood cell deformability [29], and rosetting [30]. Other complications of the disease might relate to a disordered inflammatory response or oxidative damage caused by plasma free hemoglobin [31]. Moreover, the likelihood that any particular *P falciparum* infection will progress to become severe or fatal probably depends on a wide range of genetic, immunological, physiological, and behavioral characteristics of both the host and the infecting parasite [30].

With specific reference to our current study, the central role played by sequestration is a question that has been long debated. Although florid sequestration of parasites in the small vessels of multiple organs is a consistent feature of postmortem studies and is supported by observations of microvascular blockage in the retina and rectal mucosa in living patients with severe falciparum malaria [32], it is likely that other pathophysiological processes also play a major role. However, the results of our current study, which mirror those from the majority of previous studies, support the conclusion that the most common and dangerous clinical complications of *P. falciparum* are directly related to the sequestered parasite load.

Most previous studies of severe malaria have been too small to investigate differences in sequestration between children with different clinical phenotypes. However, the relatively large sample size allowed this in our current study. We found that the SI was high across a range of different phenotypes but particularly high in severe malarial anemia. As with most complications, the etiology of severe anemia is multifactorial, involving both hemolysis and an inappropriately low erythropoietic response [33]. Nevertheless, our findings suggest that such processes may also be related to parasite load.

The children with “uncomplicated” malaria who we recruited to our current study were significantly sicker than those in many previous studies, including the Tanzanian study described above [26], because we enrolled children from a hospital as opposed to an outpatient setting. This may explain why a surprising proportion had a *Pf*HRP2 value of >1000 ng/mL, a value that has been proposed as a threshold for identifying children with “true” severe malaria [6, 26]. The study by Rubach et al [9] also found that a substantial proportion of children with uncomplicated malaria had a plasma *Pf*HRP2 of >1000 ng/mL, again perhaps reflecting a sicker population, although the median level was still less than half that in children with severe malaria.

In our current study there was considerable overlap in plasma *Pf*HRP2 concentrations between the uncomplicated and severe malaria groups, but the SI was more discriminatory, being approximately 8-fold higher in the severe than in the uncomplicated malaria group, in which the SI was similar to that in the group with asymptomatic malaria. The SI of approximately 40 is close to estimates based on the postmortem examination of brain tissue from Southeast Asian patients with cerebral malaria [34, 35]. The low predictive value of peripheral parasite densities is a consistent finding in previous studies [10, 26]. Also of relevance is the proportion of mature-stage parasites, consistent with the hypothesis that this also reflects the total parasite biomass and thus the severity of the disease [36]. Unfortunately, peripheral blood parasites were not staged in the current study.

Although a positive correlation between plasma *Pf*HRP2 and malaria severity has been found in the majority of studies, this has not been universal. In one study from Papua New Guinea, no significant difference in *Pf*HRP2 was found between children with severe versus uncomplicated malaria [13]. In agreement with observations from elsewhere in the Pacific [37], case fatality was very low in that study, and *Pf*HRP2 concentrations in children defined as having severe malaria were considerably lower than in other studies. The same profile of low mortality rate and low *Pf*HRP2 concentrations was reported in a second study, conducted in the Gambia [14], in which the authors also found no correlation between plasma *Pf*HRP2 and severity. The lack of agreement between these studies and our own might thus be explained by different definitions for severe malaria.

There are 2 potential caveats to the use of *Pf*HRP2 levels for predicting prognosis and directing care to those at greatest risk. First is the recent recognition that deletions in the genes encoding *pfhrp2* (and its homolog *pfhrp3)* mean that some clones of *P. falciparum* parasites do not express *Pf*HRP2 [38]. Although the existence of such parasites is now well established, at present they have only been found in high proportions in Latin America and the horn of Africa [39, 40]; rates of >40% have been detected in a number of studies in the Peruvian Amazon. Rates >2% are rare among studies from sub-Saharan Africa [38] (eg, Ghana [41] and Rwanda [42]). This increasing trend toward the presence of *pfhrp2* deletions could undermine the utility of quantitative *Pf*HRP2 levels going forward. Furthermore, our observations cannot be extended to nonfalciparum infections, which are common in parts of Ethiopia and Eritrea [40].

The second caveat relates to the recognition that some individuals produce antibodies to *Pf*HRP2 that could potentially reduce the levels measurable in plasma [43]. Although such antibodies are common in some settings, the degree to which they suppress plasma levels is yet to be determined [43]. In the current study, *Pf*HRP2 was undetectable despite the presence of *P. falciparum* parasites in peripheral blood in only 1.7% of the children, suggesting that despite the above caveats, *Pf*HRP2-based risk-assessment methods remain currently useful.

In summary, we have found that the *P. falciparum* parasite load, as estimated through measurement of plasma *Pf*HRP2, is strongly related to the severity of clinical malaria in children on the Kenyan coast, and that a low plasma *Pf*HRP2 level suggests an alternative pathological mechanism. This does not mean that severely ill children with a positive blood film result but a low *Pf*HRP2 level should be denied appropriate antimalarial treatment, nor that antibiotics should be withheld from those with a high *Pf*HRP2 level. Both should be treated empirically, as currently recommended in the World Health Organization guidelines [18], but clinicians should be alert to the potential for an alternative diagnosis. Our observation adds weight to the hypothesis that treatments that reduce sequestration, such as the heparinlike molecule sevuparin [44], might be useful in mitigating or reversing disease severity in patients infected with *P falciparum* parasites.

## Supplementary Material

Supplementary materials are available at Clinical Infectious Diseases online. Consisting of data provided by the authors to benefit the reader, the posted materials are not copyedited and are the sole responsibility of the authors, so questions or comments should be addressed to the corresponding author.

Supplementary Materials

## Figures and Tables

**Table 1:** Demographic and Clinical Characteristics of Children with Malaria, Stratified by Severity Grouping

	Severe Malaria^a^								
Characteristics	All severe malaria patients	CM	SMA	RD	Other	Uncomplicated Malaria	*P* Value^b^	Asymptomatic Malaria	*P* Value^c^
Total, no. (%)	1521 (100)	881 (58.0)	335 (22.0)	477 (31.4)	330 (21.7)	198 (100)	NA	28 (100)	NA
Female sex, no. (%)	753 (49.5)	442 (50.2)	171 (51.0)	232 (48.6)	154 (46.7)	92 (46.5)	<.001	7 (35.0)	<.001
Age, (median, IQR), y	2.4 (1.4–3.7)	2.3 (1.4–3.6)	1.7 (0.8–2.5)	2.2 (1.3–3.3)	2.8 (1.8–4.7)	3.1 (1.9–4.3)	<.001	7.6 (6.5–9.5)	<.001
WAZ score, mean (SD)	–1.9 (1.3)	–1.9 (1.3)	–2.1 (1.2)	–1.9 (1.4)	–1.7 (1.2)	–1.7 (0.9)	.12	NA	NA
HAZ score, mean (SD)	–1.4 (1.8)	–1.3 (1.7)	–1.5 (1.8)	–1.4 (1.9)	–1.4 (1.7)	–1.5 (1.2)	.53	NA	NA
Prostration (BCS score, 3 or 4), no. (%)	271/1484 (18.3)	0/881 (0)	36/317 (11.4)	89/472 (18.9)	169/315 (53.7)	0(0)	<.001	NA	NA
Coma (BCS score <3), no. (%)	881/1484 (59.4)	881/881 (100)	151/317 (47.6)	317/472 (67.2)	0/315 (0)	0(0)	<.001	NA	NA
Hemoglobin, mean (SD), g/dl_	7.0 (2.5)	7.2 (2.4)	3.7 (0.9)	6.8 (2.6)	7.8 (2.0)	8.9 (1.6)	<.001	11.3 (1.2)	<.001
Hematocrit, mean (SD), %	22.1 (7.8)	23.0 (7.5)	11.8 (2.8)	21.4 (8.0)	24.6 (6.3)	27.5 (4.7)	<.001	NA	NA
Severe anemia (Hb <5 g/dl_), no. (%)	335 (22.0)	151 (17.1)	335 (100)	122 (25.3)	0(0)	0(0)	<.001	NA	NA
RD, no. (%)	477 (31.5)	317 (36.2)	122 (36.4)	477 (100)	0(0)	50 (25.1)	<.001	NA	NA
Hypoglycemia, no. (%)	187 (12.3)	126 (14.3)	59 (17.6)	95 (19.9)	13 (3.9)	8 (4.0)	<.001	NA	NA
Base deficit, mean (SD), mmol/L	–10.5 (6.2)	–10.6 (5.9)	–13.1 (7.0)	–13.4 (6.4)	–7.5 (5.4)	–4.1 (4.1)	<.001	NA	NA
Severe acidosis (BD <8 mmol/L), no (%)	766/1238 (61.8)	471/720 (65.4)	196/260 (75.3)	319/400 (79.7)	111/275 (40.3)	24/176 (13.6)	<.001	NA	NA
IBI, no. (%)^d^	50 (3.3)	26 (2.9)	15 (4.5)	10 (2.1)	12 (3.6)	1 (0.5)	.02	NA	NA
Hyperparasitemia, no. (%)	454 (29.8)	266 (30.0)	80 (23.8)	169 (35.4)	93 (28.1)	79 (39.9)	.004	NA	NA
In-hospital death, no. (%)	178 (11.7)	128 (14.5)	47 (14.0)	79 (16.5)	16 (4.8)	0(0)	<.001	NA	NA

Abbreviations: BCS, Blantyre coma scale; CM, cerebral malaria; HAZ score, height-for-age z score; Hb, hemoglobin; I Bl, invasive bacterial infection; IQR, interquartile range; NA, not available or not applicable; RD, respiratory distress; SD, standard deviation; SMA, severe malarial anemia; WAZ score, weight-for-age z score.

a Some children with severe malaria manifest >1 clinical feature of severity.

b Between uncomplicated and all severe malaria.

c Between asymptomatic and all severe malaria.

d The following organisms grew in cultures from patients with severe malaria: *Enterobacter cloacae* (n = 1), *Escherichia coll* (n = 4), *Haemophilus influenzae* (n = 3), *Neisseria species (¤ =* 1), *Pseudomonas species (¤* =4), *Shigella sonnet* (n = 1), nontyphoidal *Salmonella species* (n = 11), *Staphylococcus aureus* (n = 4), group A *Streptococcus* (n = 4), *Streptococcus pneumoniae* (n = 16), and *Streptococcus viridans* (n = 1). S. *viridanswas* cultured from a patient with uncomplicated malaria (n = 1). All IBI organisms were grown from blood cultures, except for a single S. *pneumoniae* infection that was detected in cerebrospinal fluid only.

**Table 2:** Quantitative Markers of Parasite Load Stratified by Severity Groupings

	Geometric Mean (95% Confidence Interval)					
			Parasite Biomass, Parasites per Child			
Patient Group	Parasite Density, Parasites/μL	P*f*HRP2, ng/ml	Total	Circulating	Sequestered	SI
Group 1: all severe malaria (n = 1521)	39 588 (34 990–44 791)	1369 (1244–1506)	1.2 × 10^12^ (1.1 × 10^12^ to 1.4 × 10^12^)	3.1 × 10^10^ (2.8 × 10^10^ to 3.6 × 10^10^)	1.2 × 10^12^ (1.1 × 10^12^ to 1.4 × 10^12^)	40.6 (35.7–46.0)
Group 2: uncomplicated malaria (n = 198)	111 064 (86 798–141 819)	843 (655–1084)	5.2 × 10^11^ (4.0 × 10^11^ to 6.8 × 10^11^)	1 1.0 × 10^11^ (8.2 × 10^10^ to 1.3 × 10^11^)	6.0 × 10^11^ (4.3 × 10^11^ to 8.4 × 10^11^)	4.9 (3.5–6.8)
Group 3: asymptomatic malaria (n = 28)	1092 (523–2280)	7 (4–12)	6.4 × 10^9^(3.9 x 10^9^to 1.0 × 10^10^)	1.5 × 10^9^ (7.4 x 10^8^ to 3.4 × 10^9^)	4.3 × 10^9^ (1.9 × 10^9^to9.4× 10^9^)	4.0 (1.8–8.8)
*P* value	<.001	<.001	<.001	<.001	<.001	<.001
Severe malaria						
Patients who survived (n = 1343)	43 840 (38 463–19 967)	1401 (1268–1547)	1.3 × 10^12^ (1.2 × 10^12^ to 1.5 × 10^12^)	3.5 × l0^10^(3.0× 10^10^ to 3.9 × 10^10^)	1.3 × 10^12^ (1.1 × 10^12^ to 1.4 × 10^12^)	38.8 (33.9–14.4)
Patients who died (n = 178)	25 737 (17 495–37 843)	1146 (833–1577)	9.4 × 10^11^ (6.7 × 10^11^ to 1.3 × 10^12^)	1.7 × 10^10^ (1.1 × 10^10^ to 2.5 × 10^10^)	1.0 × 10^12^ (7.2 × 10^11^ to 1.4 × 10^12^)	56.9 (39.4–82.3)
P value	.01	.33	.052	<.001	.10	.01

Abbreviations: P/HRP2, *Plasmodium falciparum* histidine-rich protein 2; SI, sequestration index.

**Table 3:** Linear Regression Analysis of Markers of Parasite Biomass Among Patients With Severe Malaria

			Adjusted Multivariate Linear Regression^a^	
Marker by Malaria Group	Patients, No.	GM Value (95% CI)	Exponentiated Coefficient (95% CI)	*P* Value
Parasite density, parasites/μL				
UM	198	111 064 (86 798–141 819)	Reference	
CM	871	39 046 (33 121–46 030)	0.38 (.26–.56)	<.001
SMA	331	40 901 (32 193–51 964)	0.35 (.24–.52)	<.001
RD	473	57 158 (46 172–70 757)	0.54 (.36–.78)	. 001
Other SM	320	32 587 (24 756–42 896)	0.28 (.19–.43)	<.001
*Pf*HRP2 (ng/ml)				
UM	198	842 (655–1084)	Reference	
CM	881	1297 (1143–1472)	1.5 (1.1–2.0)	.006
SMA	335	2565 (2137–3078)	3.2 (2.3–4.5)	<.001
RD	477	1728 (1466–2037)	2.1 (1.5–2.8)	<.001
Other SM	330	1026 (829–1270)	1.1 (.9–1.6	.46
Total biomass parasites/child				
UM	191	5.2 × 10^11^ (4.0 × 10^11^ to 6.8 × 10^11^)	Reference	
CM	876	1.2 × 10^12^ (1.0 × 10^12^ to 1.4 × 10^12^)	2.2 (1.6–3.0)	<.001
SMA	335	2.3 × 10^12^ (1.9 × 10^12^ to 2.8 × 10^12^)	4.7 (3.3–6.5)	<.001
RD	473	1.5 × 10^12^ (1.3 × 10^12^ to 1.8 × 10^12^)	2.9 (2.1–4.1)	<.001
Other SM	328	1.1 × 10^12^ (8.5 × 10^11^ to 1.3 × 10^12)^	1.9 (1.3–2.7)	.001
SI				
UM	191	4.9 (3.5–6.8)	Reference	
CM	875	38.7 (32.6–46.0)	72 (4.8–11.0)	<.001
SMA	335	82.4 (64.5–105)	18.2 (11.8–28.2)	<.001
RD	473	36.1 (28.6–45.5)	74 (4.8–11.2)	<.001
Other SM	327	35.8 (27.4–46.7)	76 (4.9–12.0)	<.001

Abbreviations: CI, confidence interval; CM, cerebral malaria; GM, geometric mean; *Pf*HRP2, *Plasmodium falciparum* histidine-rich protein 2; RD, respiratory distress; SI, sequestration index; SM, severe malaria; SMA, severe malarial anemia; UM, uncomplicated malaria.

a Linear regression analysis was adjusted for Hemoglobin S genotype (HbS), α-thalassemia genotype, and ethnicity. Values show the exponentiated coefficients of the log_10_-transformed data.
