# Supplementary material for: Plasma Plasmodium falciparum Histidine-rich Protein 2 Concentrations in Children With Malaria Infections of Differing Severity in Kilifi, Kenya
Source: Clin Infect Dis. Author manuscript; Available in PMC 2021 Oct 11. (PMC8492128; doi:10.1093/cid/ciaa1141)
Supplement: Supplementary Materials [file EMS124244-supplement-Supplementary_Materials.docx]

**Figure S1.** Study flow.

**Group 3**

5 PfHRP2 undetectable

2 PfHRP2 undetectable

23 PfHRP2 undetectable

653 children with no plasma available

33 plasma samples available

28 plasma samples available

33 children <14 years who were members of a cohort study, with asymptomatic *P. falciparum* malaria detected through cross-sectional surveys during 2010 and 2011

198 plasma samples available

1,521 plasma samples available

200 plasma samples available

200 children admitted to KCH with uncomplicated *P. falciparum* malaria between 2004 and 2005

1,544 plasma samples available

**Group 2**

**Group 1**

2,197 children <14years presenting to KCH with features of severe *P. falciparum* malaria between 1998 and 2010
